# FLT1 and transcriptome-wide polyadenylation site (PAS) analysis in preeclampsia

**DOI:** 10.1038/s41598-017-11639-6

**Published:** 2017-09-22

**Authors:** Ami Ashar-Patel, Yasin Kaymaz, Augustine Rajakumar, Jeffrey A. Bailey, S. Ananth Karumanchi, Melissa J. Moore

**Affiliations:** 1 RNA Therapeutics Institute, University of Massachusetts Medical School, Worcester, MA USA; 2 Program in Bioinformatics and Integrative Biology, University of Massachusetts Medical School, Boston, MA USA; 3 Division of Transfusion Medicine, Department of Medicine, University of Massachusetts Medical School, Worcester, MA USA; 4 Departments of Gynecology and Obstetrics, Emory University, Atlanta, USA; 5 Departments of Medicine, Obstetrics and Gynecology and Center for Vascular Biology Research, Beth Israel Deaconess Medical Center and Harvard Medical School, Boston, MA USA

## Abstract

Maternal symptoms of preeclampsia (PE) are primarily driven by excess anti-angiogenic factors originating from the placenta. Chief among these are soluble Flt1 proteins (sFlt1s) produced from alternatively polyadenylated mRNA isoforms. Here we used polyadenylation site sequencing (PAS-Seq) of RNA from normal and PE human placentae to interrogate transcriptome-wide gene expression and alternative polyadenylation signatures associated with early-onset PE (EO-PE; symptom onset < 34 weeks) and late-onset PE (LO-PE; symptom onset > 34 weeks) cohorts. While we observed no general shift in alternative polyadenylation associated with PE, the EO-PE and LO-PE cohorts do exhibit gene expression profiles distinct from both each other and from normal placentae. The only two genes upregulated across all transcriptome-wide PE analyses to date (microarray, RNA-Seq and PAS-Seq) are NRIP1 (RIP140), a transcriptional co-regulator linked to metabolic syndromes associated with obesity, and Flt1. Consistent with sFlt1 overproduction being a significant driver of clinical symptoms, placental Flt1 mRNA levels strongly correlate with maternal blood pressure. For Flt1, just three mRNA isoforms account for > 94% of all transcripts, with increased transcription of the entire locus driving Flt1 upregulation in both EO-PE and LO-PE. These three isoforms thus represent potential targets for therapeutic RNA interference (RNAi) in both early and late presentations.

## Introduction

Preeclampsia (PE) is one of the three leading causes of premature birth^1^. Characterized by hypertension and proteinuria in the third trimester, PE complicates up to 10% of pregnancies, and is estimated to result in more than 76,000 maternal and 500,000 infant deaths each year worldwide (www.preeclampsia.org). In the developed world, significant resources are dedicated to the identification and management of preeclampsia, with US health care costs estimated to be 40–100 times higher for early-onset preeclamptic deliveries than for uncomplicated term deliveries^2^. PE is a highly heterogeneous disease that initiates in the placenta and is likely driven by multiple mechanisms^3,4^. In particular, early onset PE (EO-PE; defined by symptom onset < 34 weeks) is thought to be clinically distinct from late onset PE (LO-PE; defined by symptom onset > 34 weeks)^5–9^. EO-PE is thought to be driven by poor placentation during early development, whereas LO-PE is considered to be a more maternal syndrome^10^.

Although the root causes of PE are not fully understood, it is now well established that the clinical manifestations are due to excess anti-angiogenic proteins, primarily “soluble fms-like tyrosine kinase 1” proteins (sFlt1s) in the mother’s bloodstream^11^. sFlt1s are truncated forms of the membrane-bound vascular endothelial growth factor (VEGF) receptor FLT1 (aka, VEGFR1) that normally function to buffer placental growth factor (PlGF) and VEGF signaling. sFlt1s contain the extracellular binding domain present in full length or membrane-bound FLT1 (mFlt1), but are secreted because they lack the transmembrane and intracellular tyrosine kinase domains. When sFlt1s are abnormally high in the mother’s circulatory system, they interfere with her body’s ability to respond to VEGF. Among other functions, VEGF is required for maintenance of the hepatic sinusoidal vasculature and other fenestrated vascular beds in the body^12^. Breakdown of these structures impairs maternal kidney function, leading to hypertension, proteinuria and cerebral edema^13–15^ – classic features of PE and eclampsia.

**Figure 1:**
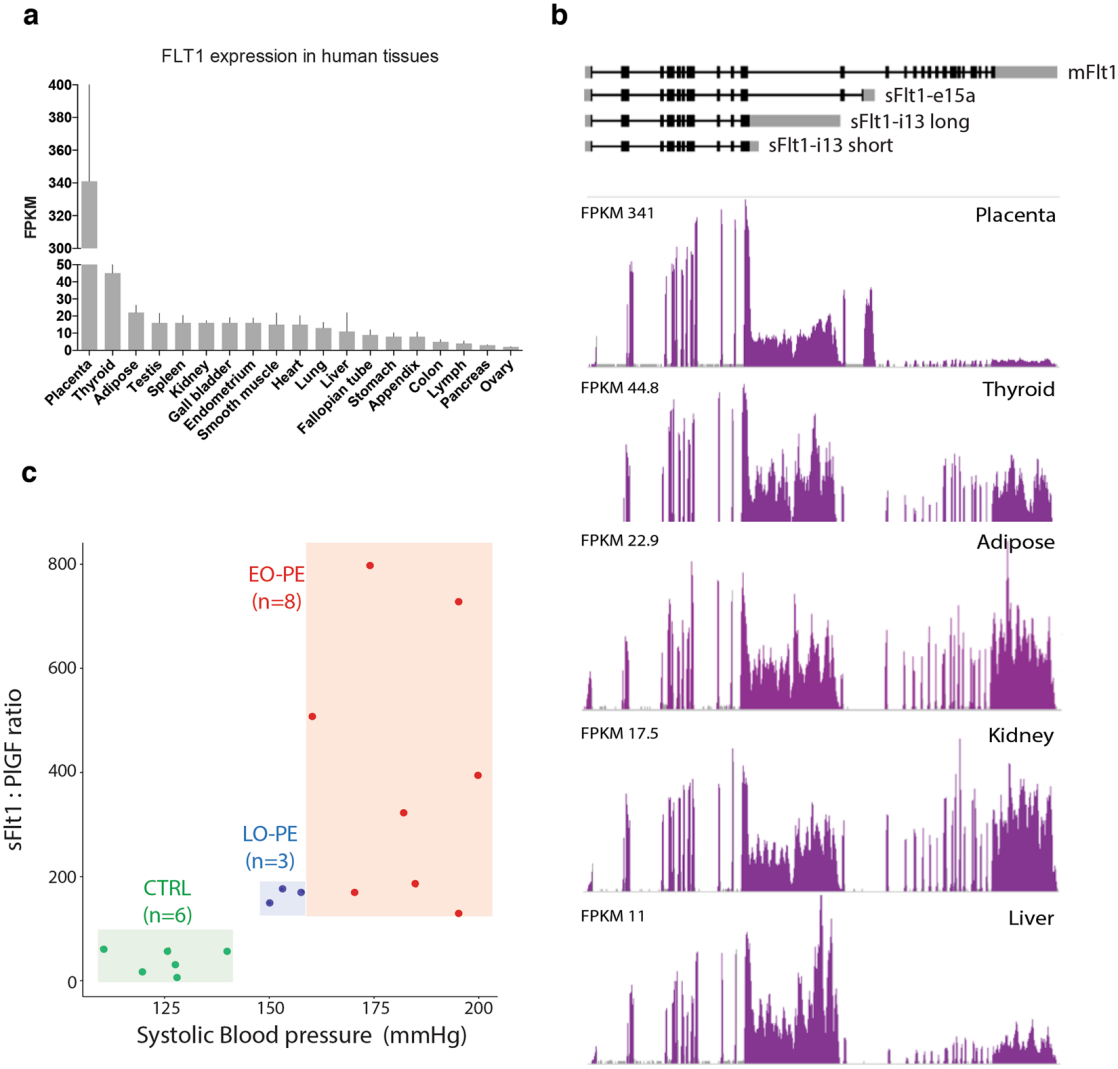
FLT1 expression and patient selection. (**a**) Mean RNA-Seq FLT1 gene expression in various human tissues. Expression values are given as Fragments Per Kilobase of transcript per Million reads (FPKM) (n = 2–7; whisker: standard deviation). (**b**) RNA-Seq data for FLT1 gene illustrating isoform differences across various tissues. All data in (**a** and **b**) are from http://v14.proteinatlas.org./ENSG00000102755-FLT1/tissue. (**c**) Patient segmentation into three groups by maternal systolic blood pressure and serum sFlt1: PlGF: Normal (CTRL, green, n = 6), Early-Onset PE (EO-PE, red, n = 8), and Late-Onset PE (LO-PE, blue, n = 3).

In mammals, FLT1 is predominantly expressed in the placenta, with human placental Flt1 mRNA levels being 40–50 times higher than those observed in any other adult tissue^16^ (Fig. 1a). Whereas the full-length isoform predominates in most adult tissues, placental and liver expression are dominated by truncated isoforms due to alternative polyadenylation (Fig. 1b). These alternative polyadenylation sites occur within introns downstream of the exons encoding the extracellular binding domain but upstream of those encoding the transmembrane and intracellular signaling domains^17–19^. Five different sFlt1 mRNA isoforms have been reported to date^20^. Two (sFlt1-i13-short and sFlt1-i13-long) result from alternative polyadenylation at different sites in intron 13 to yield mRNAs encoding the same 687 amino acid sFlt1 protein isoform, but with either a 17 or 4146 nt 3′-UTR(Untranslated region), respectively. Three others (sFlt1-i14, sFlt1-e15a and sFlt1-e15b) are due to alternative polyadenylation within intron 14. sFlt1-e15a and sFlt1-e15b result from activation of two different cryptic splice acceptors (cryptic 3′ splice sites) upon use of alternative polyadenylation sites in the latter half of intron 14. In all, these five sFlt1 mRNAs encode four different sFlt1 proteins with different C-termini.

In women with PE, circulating sFlt1 protein levels are 5 to 7 times higher than those observed in normal pregnancies^20–22^. One potential therapeutic approach would be to selectively knock down sFlt1 expression by RNA silencing (RNA interference, RNAi) using short interfering RNAs (siRNAs) targeting sequences unique to the sFlt1 mRNA isoforms and not contained in mFlt1 mRNA (manuscript in preparation). Success of this strategy requires knowing exactly which sFlt1 mRNA isoforms to target. Further, while circulating sFlt1 levels increase in both EO-PE and LO-PE, this increase starts earlier in EO-PE and often with a more aggressive trajectory^23^. This raises the question of whether EO-PE placentae express a different pattern of sFlt1 mRNA isoforms than LO-PE placentae. That is, is it possible to define a common set of sFlt1 mRNA isoforms suitable for therapeutic intervention in both EO-PE and LO-PE patients?

Here we applied polyadenylation site sequencing (PAS-Seq) method on RNA isolated from normal (CTRL) and PE human placentas to identify the most abundant sFlt1 mRNA isoforms. Because PAS-Seq specifically interrogates polyadenylation sites it provides a much more accurate assessment of transcript 3′ ends than RNA-Seq^24–26^, which is generally biased toward transcript 5′ ends and is a better for investigating alternative splicing pattern^27,28^. Like RNA-Seq, PAS-Seq can be used for differential gene expression analysis by combining all isoforms detected at an individual gene locus into a single gene expression number^24^. Therefore, in addition to quantifying alternative polyadenylation both at the Flt1 locus and transcriptome-wide, our PAS-Seq data also allowed us to investigate overall gene expression changes between our patient groups.

## Results

### Placental PAS-Seq datasets

Several risk factors are associated with PE^29–31^. In an attempt to minimize confounding variables by other associated conditions, we used highly selective criteria for patient inclusion. First, we limited our sample set to singleton pregnancies, and excluded any patients with preexisting hypertension or diabetes mellitus. Second, all of our control set (CTRL; n = 6) delivered at term with no other pregnancy complications (such as preterm labor or premature rupture of membranes). Finally, we limited our PE set (n = 11) to patients with systolic blood pressures ≥ 140 mm Hg and circulating sFlt1:PlGF ratios ≥ 85, an accepted diagnostic measure of PE associated with adverse PE related maternal/fetal outcomes^11,32,33^. (Fig. 1c and Table 1). Gestational delivery week, ranged from 37 to 40 weeks in the CTRL set and from 28 to 39 weeks in the PE set. Based on the gestational delivery week, we further divided the PE patients into Early-Onset (EO-PE; gestational delivery week < 34; n = 8) and Late-Onset (LO-PE; gestational delivery week > 34; n = 3) subgroups. In all cases, placental tissue was collected within 30 minutes of delivery and flash frozen in liquid nitrogen. Using these frozen samples, we isolated polyA tailed RNA from the villous tissue, which is the spongy layer in between the decidua (maternal side) and the chorion (fetal side), and the main source of sFlt1^34^.

**Table 1:** PAS-Seq patient characteristics.

Variables	CTRL (n = 6)	EO-PE (n = 8)	LO-PE (n = 3)
Systolic BP (mmHg)	125 ± 10	183 ± 14***	154 ± 4**
Diastolic BP (mmHg)	79 ± 8	109 ± 11**	98 ± 15
Maternal plasma sFlt1 (pg/mL)	9238 ± 4257	29672 ± 10420***	26243 ± 13805*
Maternal plasma PGF (ng/mL)	331 ± 262	98 ± 55*	158 ± 77
sFlt1: PlGF ratio	40 ± 24	405 ± 256***	166 ± 14*
Maternal age (years)	35 ± 8	31 ± 5	27 ± 6
Maternal pre-pregnancy BMI (kg/m^2^)	26 ± 5	34 ± 5*	18 ± 8
Gestational delivery week	39 ± 1	31 ± 2***	37 ± 2
Proteinuria	NA	100%	100%
Newborn gender (F/M)	3/3	4/4	2/1
Mode of delivery (C-sec/Vaginal)	6/0	8/0	3/0
Induction of labor (yes/no)	0/6	1/7	2/1

Mean statistic ± standard deviation. Patient set comparisons were performed using the Mann-Whitney test; *P = 0.05, **P = 0.001, ***P = 0.0002.

**Figure 2:**
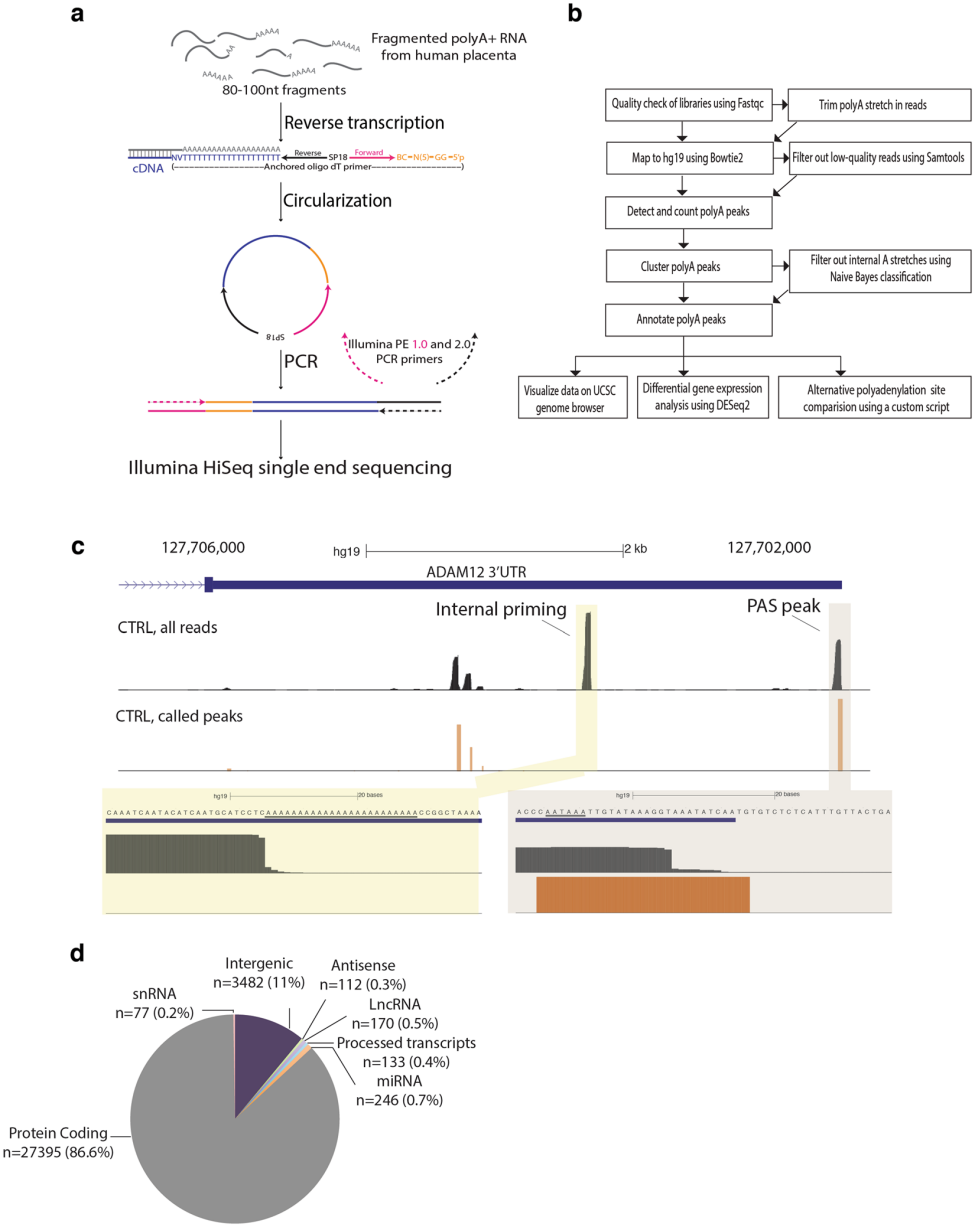
PAS-Seq library and bioinformatics workflow. (**a)** Schematic overview of PAS-Seq library preparation workflow. (**b)**. PAS-Seq bioinformatics pipeline schematic. (**c)** Representative view of read alignment locations (top track) and called PAS peaks (bottom track) on the ADAM12 3′-UTR. Our algorithm is able to remove background noise, filter out internal priming sites and accurately identify true polyadenylation sites. (**d**) Distribution of PAS peaks among named gene classes and intergenic regions.

To analyze alternative polyadenylation transcriptome-wide, we developed a simple, kit-free method by combining elements of a published PAS-Seq protocol^25^ with a circularization strategy used in our lab for making short RNA fragment libraries^35^ (Fig. 2a, see Methods). Single-end sequencing on the Illumina HiSeq. 2000 platform yielded 30–70 million (in average) high quality reads per library, of which 70–85% mapped uniquely to the human genome (hg19) (Supplementary Table S1). Using a custom pipeline (Fig. 2b and Methods), peaks were identified as clusters of ≥ 15 reads (all libraries combined) whose 3′ ends mapped within a 40 nt window (to allow for microheterogeneity in polyadenylation site use)^36^. Peaks representing true polyadenylation site (i.e., those at which a polyA tail was added post-transcriptionally) were distinguished from peaks due to oligoT internal priming of transcripts at genomically-encoded adenosine-rich regions using a Naives Bayes classifier based probabilistic method^25^. True polyadenylation site were then assigned to genes using GENCODE (v19) annotations and quantified as counts per million (CPM). Custom UCSC genome browser tracks enabled visualization of read clusters and true polyadenylation site sites (Fig. 2c). In all, we detected 31,615 polyadenylation sites on or within 5 kb downstream of annotated transcripts representing 13,328 genes and 3,482 polyadenylation sites in intergenic regions. The majority of the annotated set was dominated by protein-coding genes (n = 27,395 PAS; 86.6%), but also contained hundreds of small nuclear RNAs, long non-coding RNAs, microRNAs and anti-sense transcripts (Fig. 2d).

**Figure 3:**
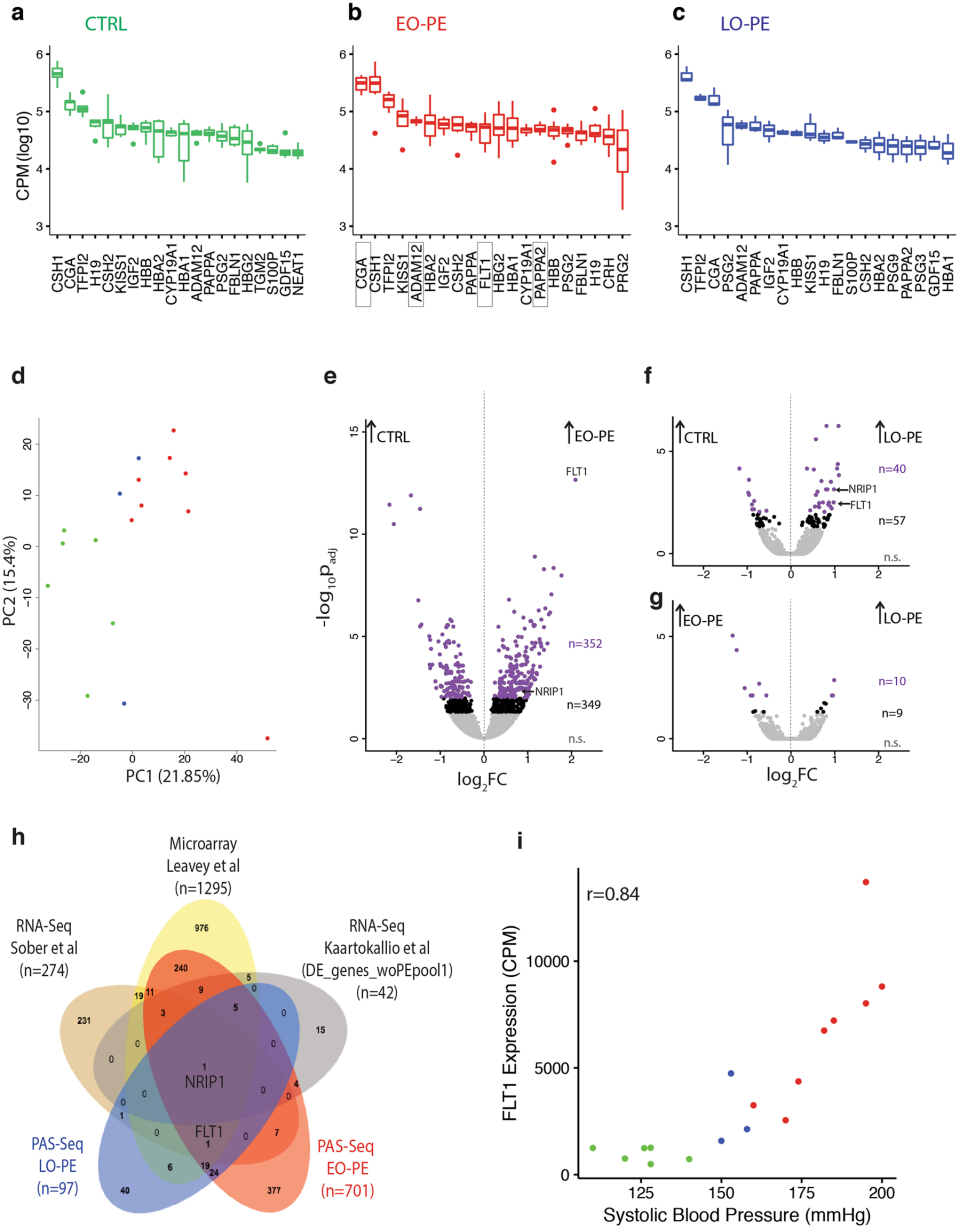
Differential gene expression analysis. Box-plots of top twenty most abundant transcripts in (**a)** CTRL, (**b)** EO-PE, and (**c**) LO-PE patients. Gene expression measured as Counts Per Million (CPM) mapped reads. Dark bar: median; box: 25–75% interquartile; whiskers and dots: range. (**d**) Principal component analysis (PCA) of highly variable genes. Axes are principal components 1 and 2 (PC1 and PC2). Each point represents a single patient, with colors indicating the patient type as in Fig. 1a. Volcano plots showing differential gene expression for (**e**) CTRL vs. EO-PE, (**f)** CTRL vs. LO-PE, and (**g)** EO-PE vs. LO-PE. X-axis: log_2_ Fold Change (FC) between samples; Y-axis: -log10 false discovery rate after false positive removal (p_adj_). Each dot is a gene: purple, p_adj_ ≤ 0.01; black, p_adj_ ≤ 0.05; n, number of genes in each group. (**h)** Venn diagram illustrating overlap of differentially expressed genes (p_adj_ cutoffs as indicated) between our EO-PE and LO-PE PAS-Seq datasets, two published RNA-Seq datasets^37,46^ and a large meta analysis of available microarray data^47^. (**i)** Scatter plot of FLT1 RNA expression (CPM) vs. Systolic Blood Pressure (mmHg). Each point represents a single patient, with colors indicating the patient type as in Fig. 1a.

By summing all true polyadenylation site reads mapping across individual loci, PAS-Seq data can be used to assess overall gene expression. That is, the data can be collapsed into a single gene expression number – counts per million mapped reads (CPM) that encompasses all polyadenylated isoforms derived from each gene^24^. The top 500 genes in our samples were highly enriched for those expressed in placenta (p ≤ 8.4 e-43) and syncytiotrophoblasts (p ≤ 6.5 e-42) (see Methods). Consistent with a previous RNA-Seq report^37^, nearly all of the 20 most highly expressed genes in the CTRL, EO-PE and LO-PE samples (Fig. 3a,b,c) were placental or pregnancy-related (Supplementary Table S2). This highest abundance set was dominated by hormones (e.g., CSH1, CSH2 and CGA), positive and negative regulators of cell growth (e.g., GDF15 and TFPI2) and hemoglobin genes (HBA1, HBA2, HBB and HBG2); also included were two high abundance long non-coding RNAs, H19 and NEAT1.

### EO-PE and LO-PE placentas exhibit distinct gene expression signatures

To determine whether the different patient sets had distinct gene expression patterns, we next performed Principal Component Analysis with all detected genes with sufficient read coverage (total read count across all 17 libraries ≥ 10; n = 15,765). By assessing variability across all samples independent of any externally imposed subsets, Principal Component Analysis (aka PCA) allows one to determine how closely related individual samples are to one another. For the top 2000 most variable genes across all libraries, Principal Component(PC) 1, 2 and 3 accounted for 21.8, 15.4 and 10.4 percent of the variance, respectively. In a plot of Principal Component 1 vs. Principal Component 2, the CTRL samples formed a coherent cluster readily distinguishable from the PE samples (Fig. 3d). Further, the EO-PE and LO-PE samples formed distinct but overlapping clusters primarily defined by PC1, with the EO-PE cluster being more distant from CTRL than the LO-PE cluster. This suggested that EO-PE and LO-PE had overlapping but distinct gene expression signatures.

To define the sets of differentially expressed genes between our three groups, we performed pairwise differential gene expression analyses between CTRL, EO-PE and LO-PE using DESeq. 2 (Fig. 3e,f,g). DESeq. 2 is a software package routinely used to analyze high-throughput PAS-Seq and RNA-Seq datasets^38^. In agreement with the Principal Component Analysis, the pair exhibiting the greatest differential expression profile was between CTRL and EO-PE (n = 701, p_adj_ ≤ 0.05). The most highly upregulated gene in EO-PE compared to CTRL was FLT1 (4.3-fold up), followed by Myosin 7B (MYO7B, 3.4-fold up) and Endoglin (ENG, 3-fold up). Other upregulated genes previously linked to PE included Inhibin Alpha (INHA, 2.8-fold up), Inhibin Beta A (INHBA, 2.5-fold up), Vascular Endothelial Growth Factor A (VEGFA, 2.2-fold up), MicroRNA 210 Host Gene (MIR210HG, 2.4-fold up) and Pappalysin 2 (PAPPA2, 2.6-fold up). Genes exhibiting the greatest downregulation were Somatostatin Receptor Type 1 (SSRT1, 4.3-fold down), Signaling Lymphocytic Activation Molecule Family Member 1 (SLAMF1, 4.1-fold down), Hyaluronan And Proteoglycan Link Protein 1 (HAPLN1, 3.2-fold down) and Cell Adhesion Molecule 3 (CADM3; 2.8-fold down).

Overall, 349 genes were differentially expressed at a p_adj_ ≤ 0.01 between CTRL and EO-PE (Fig. 3e). Using a variety of online tools such as GeneCoDis^39–41^ and Genomatix, we performed extensive gene ontology analyses of this set, using all other expressed 13,299 genes as background (Supplementary Table S3). Recurring themes (Supplementary Tables S4 and S5) were Biological/Cellular Adhesion and the HIF1 alpha, HIF2 alpha, Interleukin 6, Angiogenesis, Cadherin, and Wnt signaling pathways, all of which have been previously linked to PE^42–44^. Also enriched were Pol II transcription factors and genes with LEF1 and TATA transcription factor binding sites (p_adj_ ≤ 0.01).

Consistent with the principal component analysis, fewer genes were differentially expressed between CTRL and LO-PE, with only 40 reaching the p_adj_ ≤ 0.01 threshold (Fig. 3f). Those exhibiting the greatest fold change in LO-PE were Perilipin 2 (PLIN2; 2.1-fold up), Zinc Finger Protein 175 (ZNF175, 2.1-fold up), Acyl-CoA Synthetase Long-Chain Family Member 1 (ACSL1, 2.1-fold up), Dual Specificity Phosphatase 15 (DUSP15, 2.3-fold down) and Forkhead Box S1 (FOXS1, 2.3-fold down). The 97 differentially expressed genes (p_adj_ ≤ 0.05; 13,265 background gene set) were highly enriched for Pol II transcription factors and components of the HIF2 alpha signaling pathway (Supplementary Tables S3, S6 and S7).

Comparison of the differentially expressed genes in EO-PE and LO-PE revealed that they have overlapping but clearly distinct gene expression profiles (Supplementary Fig. S2). Gene ontology terms associated with the 50 differentially expressed genes common to both EO-PE and LO-PE (p_adj_ ≤ 0.05) includes the HIF1 alpha, HIF2 alpha and VEGFR1 (FLT1) signaling pathways (Supplementary Table S8). Differential expression analysis between EO-PE and LO-PE sets revealed 19 genes reaching the p_adj_ ≤ 0.05 threshold (Fig. 3g), with the genes exhibiting the greatest differences being Family With Sequence Similarity 19 Member A2 (FAM19A2, 2.5-fold higher in EO-PE), Pleiomorphic Adenoma Gene-Like 2 (PLAGL2, 2.3-fold higher in EO-PE), TNF Receptor Superfamily Member 10a (TNFRSF10A, 1.9-fold higher in LO-PE) and HAPLN1 (1.9-fold higher in LO-PE). Thus, while EO-PE and LO-PE have similar clinical presentations, their distinct gene expression signatures suggest different mechanistic drivers^9,10,45^.

### Comparison to previous differential gene expression studies

Numerous other studies have employed microarrays or RNA-Seq to investigate differential gene expression in PE. We therefore compared our differentially-expressed EO-PE and LO-PE gene sets (p_adj_ ≤ 0.05) to similar gene sets from two recent RNA-Seq studies^37,46^ and a large scale meta analysis of microarray datasets^47^ (Fig. 3h). As expected from the heterogeneous and multifactorial nature of PE, the gene sets reported in the three previous studies were largely non-overlapping (Fig. 3h). Our gene lists were most similar to the microarray list^47^, with 289 of 701 (41%) differentially-expressed EO-PE genes and 32 of 97 (33%) differentially-expressed LO-PE genes being among the 1295 genes (p_adj_ ≤ 0.01) in the microarray set. Notably, only one gene, NRIP1 (nuclear receptor-interacting protein 1; aka RIP140), intersected all five lists (Fig. 3h). Upregulated in all datasets, NRIP1 is a ubiquitously-expressed nuclear protein that acts as a co-activator and/or co-repressor for numerous nuclear receptor transcription factors^48^.

The only other differentially expressed gene reported in all previous studies and ours was FLT1. Although FLT1 did not appear in the list of differentially expressed genes in the Kaartokallio *et al*. study^46^, it was stated in the text as being differentially expressed. The magnitude of FLT1 overexpression in PE varies greatly among published reports – whereas the microarray and RNA-Seq values ranged from 1.5 to 2.0-fold^37,47^, qPCR analyses specifically interrogating the FLT1 locus range from 3.86-fold to 4.5-fold above normal^16,37^. In our PAS-Seq data, FLT1 was the most highly upregulated gene in the EO-PE patient set, exhibiting a 4.3-fold increase relative to the CTRL set (p_adj_ = 2.21E-13; Fig. 3e). FLT1 was also upregulated the LO-PE set, but only by 1.9-fold (p_adj_ ≤ 0.01; Fig. 3f). Further, consistent with Flt1 overexpression being a significant driver of clinical symptoms, total Flt1 gene expression strongly correlated with systolic blood pressure over all samples (r = 0.84) (Fig. 3i). Flt1 also correlated with body mass index (BMI), but to a much lesser degree (r = 0.51, Supplementary Fig. S2). Thus our PAS-Seq data validate previous findings that Flt1 overexpression is central to PE.

### Non-coding RNAs and intergenic regions

In addition to protein-coding genes, our PAS-Seq data also provided expression values for numerous non-coding RNAs (Supplementary Table S3). For example, many miRNAs (miRs) derive from long non-coding polyA + primary transcripts (pri-miRNAs)^49^. Pri-miRNAs exhibiting particularly high placental expression include the chromosome 14 and 19 miR clusters (C14MC and C19MC, respectively), the miR-371–3 cluster and the mir210 host gene (mir210HG)^50–52^. C19MC is transcribed by Pol III^53^, so was not represented in our PAS-Seq data. Of the remaining three, only the mir210HG exhibited differential expression among our patient sets, being 2.4 and 1.8-fold increased in EO-PE and LO-PE, respectively, relative to CTRL. Previously shown to be upregulated in PE^54^, miR210 is known to downregulate the THSD7A gene^55^. Consistent with this, THSD7A was 1.5-fold downregulated in EO-PE (p_adj_ ≤ 0.043), and 1.3-fold downregulated in LO-PE (although not statistically significant; p_adj_ ≤ 0.58).

Other differentially expressed noncoding transcripts with embedded miRNAs were DNM3OS (miR214; 1.7-fold down in both EO-PE and LO-PE) and RP6–99M1.2 (miR221/miR222; 1.6-fold down in EO-PE). Finally, some miRNAs derive from the introns or 3′-UTRs of protein coding genes, including SREBF1 (miR33b and miR6777; 1.6-fold up in EO-PE, 1.5-fold up in LO-PE), PDCD4 (miR4680; 1.5-fold up in EO-PE), and FAM172A (miR2277; 1.4-fold down in EO-PE). Among this set, only miR214 has been previously linked to PE, with microarray data indicating its downregulation^56^. miR214 is thought to regulate both PLGF^57^ and components of the β-catenin pathway^58^, with its own expression being modulated by HIF1 alpha^57^.

Our datasets also included polyadenylation sites for 170 long non-coding RNAs, 112 anti-sense transcripts and 133 “processed transcripts” (defined in GenCode as a transcript with no open reading frame). Although nine of these were differentially expressed in EO-PE, LO-PE or both (Supplementary Table S3), careful examination of the associated polyadenylation site peaks on the UCSC genome browser revealed that nearly all could be explained by differential expression of an overlapping protein coding gene. Thus we conclude that differential long non-coding RNA expression is not a general hallmark of PE. The sole exception was TP73-AS1 (downstream of and antisense to TP73, expression of which was undetectable in our dataset), a lowly expressed transcript (base mean = 62.5 CPM) that was 1.4 fold-downregulated (p_adj_ = 0.02) in EO-PE. TP73-AS1 is differentially expressed in multiple cancers^59^, and recent evidence suggests that it functions to positively regulate expression of BDH2, a cytosolic type 2 hydroxybutyrate dehydrogenase involved in mitochondrial function^59^. While BDH2 expression was 1.4-fold reduced in our EO-PE set, this change was not statistically significant (p_adj_ = 0.15).

Finally, we examined the 3,482 intergenic polyadenylation sites. Compared to the mean CPM distribution for genic PAS, the vast majority of intergenic PAS were lowly expressed (Supplementary Fig. S1), and DESeq. 2 analysis yielded only 13 and 8 differentially expressed intergenic polyadenylation site in EO-PE and LO-PE, respectively (Supplementary Table S9). Many of these were associated with Long Interspersed Elements (LINEs) and Long Terminal Repeat elements (LTRs), suggesting that activation of select endogenous retroviral elements might occur in PE. For example, three intergenic PAS upregulated in EO-PE lie between the LTF and CCRL2 genes in a region containing numerous retroviral elements, and an RNA-Seq track of multimapping reads confirmed the existence of an independent multiple LTR-containing transcript (Supplementary Fig. S1).

### All Flt1 isoforms are upregulated in PE

**Figure 4:**
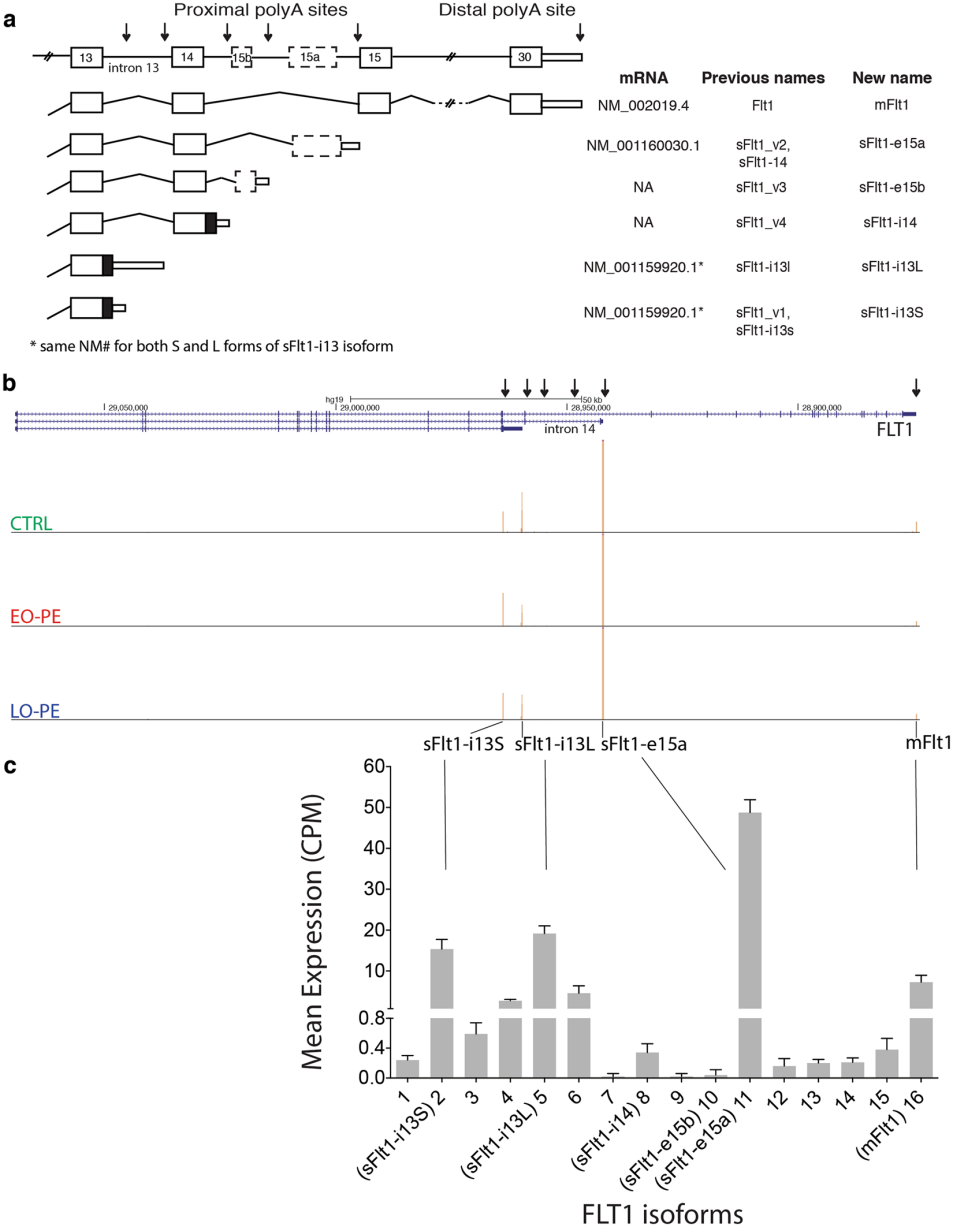
FLT1 alternative PAS isoform analysis. (**a**) Schematic of FLT1 gene and six previously identified mRNA isoforms arising from alternative polyadenylation. Thick boxes: protein coding exons; thin boxes: 3′-UTRs; lines: introns; arrows: sFlt1 and mFlt1 polyadenylation sites. Table shows all previous isoform names and new systematic names. (**b**) UCSC genome browser view showing PAS-Seq peak distribution on the FLT1 gene for a representative CTRL (top track), EO-PE (middle-track) or LO-PE (bottom track) patient. Arrows: sFlt1 and mFlt1 polyadenylation sites for isoforms in (**a**). (**c**) Bar graph showing mean expression (counts per million; CPM) across all libraries for all 16 detected mRNA isoforms at the FLT1 locus.

A major goal of this study was to determine which sFlt1 mRNA isoform(s) to target with therapeutic siRNAs^60^ (Turanov *et al*., in preparation). Our PAS-Seq data revealed a total of 16 PAS in the Flt1 gene (Fig. 4b and c; Supplementary Table S10), including all 6 previously reported Flt1 mRNA isoforms. Thirteen are low abundance, each representing < 0.6% of true PAS reads mapping to the Flt1 locus. Two previously reported isoforms, sFlt1-i14 (previously sFlt1-v4) and sFlt1-e15b (previously sFlt1-v3), were among this low abundance set, so contribute little to sFlt1 expression. The four high abundance species are sFlt1-i13S (previously sFlt1_v1 short 3′UTR), sFlt1-i13L (previously sFlt1_v1 long 3′UTR), sFlt1-e15a (previously sFlt1_v2) and m-Flt1 (membrane-bound or full-length Flt1 mRNA) (Fig. 4a). Among these, sFlt1-e15a is the most abundant, accounting for ~50% of Flt1 PAS reads in all samples (Fig. 4b). The sFlt1-i13 short and long isoforms each accounted for 20 to 25% of PAS reads, whereas full-length m-Flt1 mRNA represented only ~5% of PAS reads. Thus, at time of delivery, sFlt1-i13 short, sFlt1-i13 long and sFlt1-e15a comprise the vast majority (88 to 97%) of placental Flt1 mRNAs.

**Figure 5:**
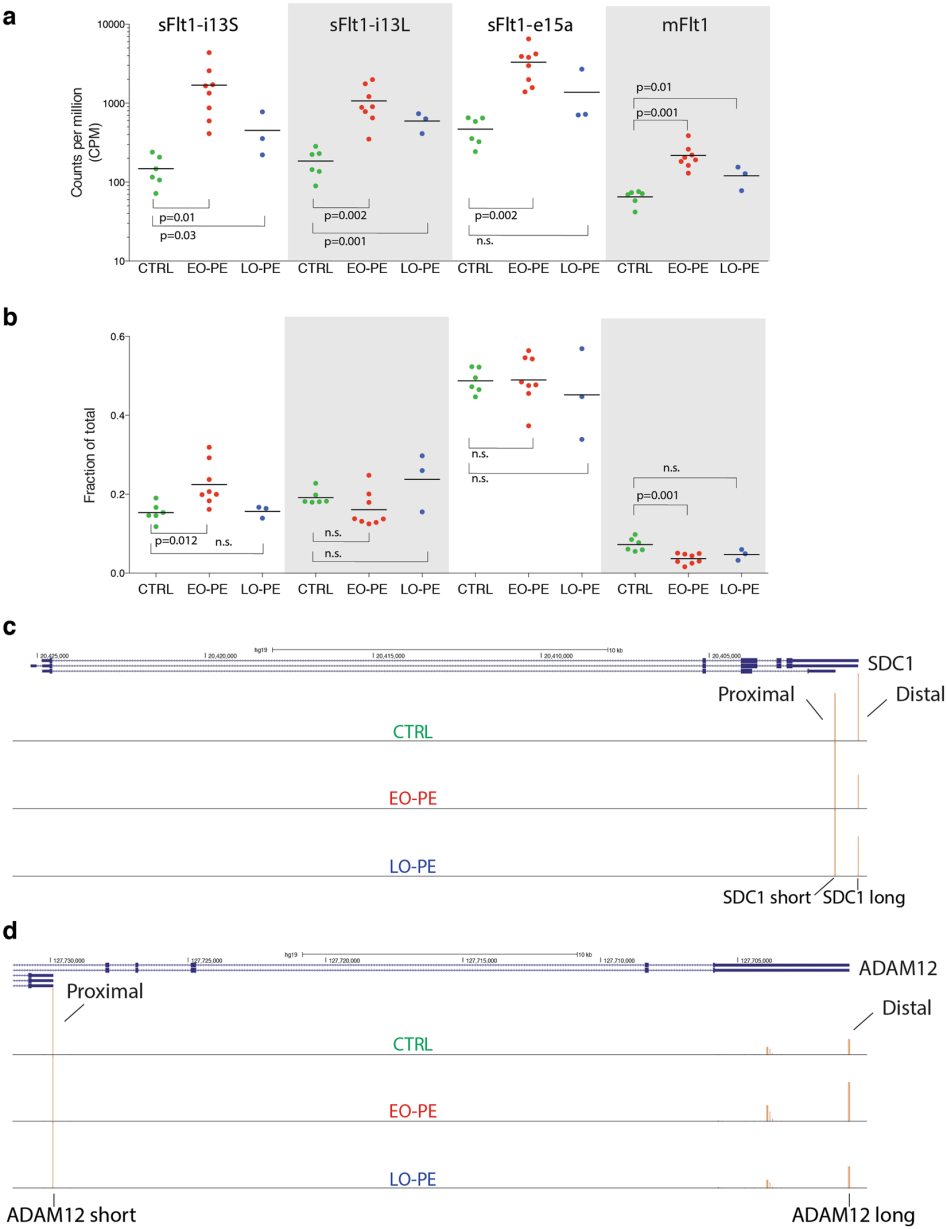
Differential PAS isoform analysis on FLT1, SDC1 and ADAM12 genes. (**a**) Superimposed scatter plots showing expression (counts per million mapped reads; CPM) of the four most abundant FLT1 mRNA isoforms. (**b**) Superimposed scatter plot comparing fractions of total PAS reads mapping to the FLT1 locus represented by each indicated mRNA isoform. For a. and b., each point represents an individual CTRL, EO-PE or LO-PE patient with the color indicating patient type as described in Fig. 1c. A two-way t-test was used for statistical comparisons between indicated groups. Horizontal line: mean; n.s.: not statistically significant. (**c**) UCSC genome browser view of SDC1 gene illustrating differential PAS isoform abundance (Distal-to-Proximal Switch) for a representative CTRL (top track), EO-PE (middle-track) or LO-PE (bottom track) patient. (**d**) Same as **c**, but ADAM12 gene (Proximal-to-Distal Switch).

Comparison among datasets revealed that all four high-abundance Flt1 isoforms are upregulated in EO-PE vs. CTRL (p ≤ 0.05; two-way t-test) (Fig. 5a). A similar trend is also observed comparing LO-PE to CTRL, but only the sFlt1-i13 long isoform reached statistical significance (p ≤ 0.05; two-way t-test), likely due to the small sample size. To assess whether this general upregulation of the Flt1 locus was accompanied by any shift in PAS usage, we examined how the fraction of total reads mapping to each of the four major PAS differed among the EO-PE, LO-PE and CTRL datasets (Fig. 5b). The only statistically significant (two-way t-test) differences were a 1.5-fold increase in sFlt1-i13 short and a 2.0-fold reduction in mFlt1 in EO-PE compared to CTRL. Thus in EO-PE, use of the sFlt1-i13 short PAS increases at the expense of the mFlt1 PAS. Nonetheless, the predominant feature associated with both EO-PE and LO-PE is transcriptional upregulation of the entire Flt1 locus.

### Other genes with altered isoform abundances

The altered Flt1 mRNA isoform abundance associated with EO-PE prompted us to ask whether this phenomenon was detectable at any other gene loci. Using one-vs.-others model (see Methods), we observed PAS switches (p_adj_ ≤ 0.05) in only eight genes in EO-PE compared to CTRL (ADAM12, FLT1, NEAT1, TIMP2, CRH, PAPPA, PSAP and SDC1) and only four genes in LO-PE compared to CTRL (EIF2A, PSG1, SDC1 and PHLDB2) (Supplementary Table S11), with SDC1 being common to both. The switches were almost equally divided between proximal-to-distal (n = 6) and distal-to-proximal (n = 5) isoform shifts. Most of the altered isoform abundances were limited to shifts within untranslated regions and so were not expected to alter protein isoform ratios. The exceptions were FLT1 (discussed above), SDC1 and ADAM12. In all three cases, the affected mRNA isoforms change the ratio of secreted and membrane-bound isoforms. The SDC1 gene encodes short (secreted or “shed”) and long (membrane-bound) forms of the major transmembrane heparan sulfate proteoglycan syndecan 1 from mRNA isoforms having different PAS (Fig. 5c). In both EO-PE and LO-PE, the isoform encoding membrane-bound SDC1 was preferentially upregulated. Likewise, ADAM12 expresses two alternate PAS mRNA isoforms, ADAM12-S and ADAM12-L, encoding short (S,secreted) and long (L,membrane-bound) forms of metalloproteinase-disintegrin 12^61,62^ (Fig. 5d). Whereas the entire gene is 1.6-fold upregulated in EO-PE, the ADAM12-L isoform increases more than the ADAM12-S isoform (1.8-fold vs. 1.3-fold, respectively).

## Discussion

Here we present comprehensive differential gene expression and PAS isoform abundance analysis of placental RNA from subjects having an abnormal plasma angiogenic profile accompanying either EO-PE or LO-PE. We find that EO-PE and LO-PE are distinct conditions with different but related gene expression signatures. Common to both is overexpression of the anti-angiogenic factor sFlt1, expression of which strongly correlates with systolic blood pressure (Fig. 3i). Our PAS-Seq analysis confirms previous reports identifying sFlt1-e15a as the predominant sFlt1 mRNA isoform in both normal and PE placentae^20,22,63–65^, with the next most abundant isoforms being sFlt1-i13 short and long^66^. Together sFlt1-i13-short, sFlt1-i13-long and sFlt1-e15a account for > 94% of all sFlt1 expression in both normal and PE placentae. This information enabled us to identify siRNAs that decrease sFlt1 expression as a potential therapeutic for PE^66^. Our observation that all Flt1 isoforms increase in PE indicates that Flt1 upregulation is primarily a transcriptional response. Intriguingly, the only other gene differentially expressed in PE across all transcriptome-wide analyses to date is the transcriptional coregulator NRIP1 (RIP140)^48,67^.

Because sFlt1 is a key negative regulator of angiogenesis in both normal^68^ and cancerous tissues^69^, how its production is modulated has been the topic of multiple studies outside the placenta. One condition known to alter sFlt1 expression is hypoxia^70,71^. In human microvascular endothelial cells (HMVECs), the predominant sFlt1 mRNA isoform is sFlt1-i13-short. Under hypoxic conditions, sFlt1-i13-short levels decrease, but mFlt1 levels are unaffected suggestive of a post-transcriptional mechanism^72^. More recent work^73^ identified heterogeneous nuclear ribonucleoprotein D (hnRNP D) as a negative regulator of sFlt1-i13-short production, possibly acting through binding to an AU-rich element near the sFlt1-i13-short PAS and thereby blocking access of the polyadenylation machinery to this location. That study further showed that this inhibitory activity was a function of both overall hnRNP D levels and the methylation status of a specific hnRNP D arginine residue. In EO-PE placental samples, however, we observed the opposite trend – a small increase in sFlt1-i13 short at the expense of mFlt1 (Fig. 5b). We also observed no differential gene expression for arginine methyltransferase (PRMT1), arginine demethylase (JMJD6), hnRNP D or any other hnRNP protein (data not shown). Thus sFlt1 upregulation in PE appears to be mechanistically unrelated to the hypoxia-dependent modulation of sFlt1-i13-short production in HMVECs.

Our data clearly show that all FLT1 mRNA isoforms increase in abundance in PE (Fig. 5a), with no general shift toward the more promoter proximal PAS (Fig. 5b). When we considered the entire transcriptome, we detected no general 3′-UTR shortening (data not shown) and only eleven statistically significant PAS isoform abundance changes overall (Supplementary Table S11 and Fig. 5c,d). Among these, there was no consistent proximal-to-distal or distal-to-proximal switch pattern. Thus there is no general polyadenylation pattern shift associated with PE, and alternative polyadenylation is not a major contributor to sFlt1 upregulation. Rather our data indicate that the increased sFlt1 expression in PE is primarily due to increased transcription of the entire FLT1 locus.

What is driving this increased FLT1 transcription? Because of its central role in regulating angiogenesis, FLT1 transcriptional regulation has been intensely investigated in other tissues and several upstream transcription factors including EPAS1, PAX3, p53 and ETS have been characterized^74–79^. Notably, the differentially expressed gene sets (p_adj_ ≤ 0.05) in both EO-PE and LO-PE included numerous Pol II transcription factors. Common to both sets were CEBPA, SP3, DLX5, BHLHE40 and SREBF1, all of which had increased expression, and ZEB2, which had decreased expression. The CEBPA (CCAAT/enhancer binding protein alpha) pathway is particularly interesting, as CEBPA is expressed in the labyrinthine trophoblasts (the same cells that express Flt1) and has been shown to regulate placental development^80^.

Intriguingly, the only other gene identified by every transcriptome-wide gene expression analysis method to date (microarray, RNA-Seq and PAS-Seq) as being differentially expressed in PE is NRIP1 (nuclear receptor-interacting protein 1; aka RIP140) (Fig. 2h). In all cases, NRIP1 was upregulated. A ubiquitously-expressed nuclear protein, NRIP1 modulates the activities of numerous nuclear receptor transcription factors^48^. Its best understood role is as a regulator of energy expenditure in adipose and muscle tissues, but it has also been linked to ovarian fertility and maintenance of a pregnancy state^81^. Whether NRIP1 contributes to the metabolic syndrome often associated with PE or it directly regulates sFlt1 expression remains to be explored. We note that NRIP1 has been implicated in the pathogenesis of obesity^48,67^a major risk factor for PE. It is possible that upregulated NRIP1 may synergize with anti-angiogenic factors to induce the endothelial dysfunction ultimately leading to PE.

Previous microarray studies comparing samples from patients with HELLP syndrome (a particularly severe form of PE associated with hemolysis, elevated liver enzymes and low platelet count) to EO-PE and/or LO-PE with CTRL patients, found that these are discrete conditions with distinct gene expression signatures and different mechanistic drivers^9,10,45^. Our PAS-Seq data are consistent with these previous findings. We found many more differentially expressed genes in the EO-PE cohort than in the LO-PE cohort, although the small size of our LO-PE dataset (n = 3) may have precluded all but the most consistently differentially expressed genes from reaching statistical significance. An additional caveat is that some gene expression differences could be due to gestational age differences, particularly between the EO-PE (gestational delivery week < 34) and CTRL (gestational delivery week >37–40) groups. Nonetheless the HIF1 alpha, HIF2 alpha and VEGFR1 (FLT1) signaling pathways were differentially expressed in both groups, consistent with hypoxia and altered angiogenesis being generally associated with PE^82–85^. But unique to the EO-PE set were genes involved in Biological/Cellular Adhesion and the Interleukin 6, Cadherin, and Wnt signaling pathways. This suggests that EO-PE placentae may have defects in epithelial to mesenchymal transition which may lead to defective trophoblast invasion noted in this syndrome^86^.

Due to its highly heterogeneous presentation, definitive diagnosis of PE based solely on maternal signs and symptoms can be erroneous^87,88^. A variety of underlying conditions can serve as mechanistic drivers, confounding gene expression analysis of diverse patient populations. Differences in sample collection methodologies across institutions can also introduce variability. These factors likely explain the relatively poor overlap among prior transcriptome-wide analyses of placental gene expression in PE (Supplementary Fig. S2). For the current study, we aimed to eliminate as many variables as possible. In addition to using highly selective criteria for PE diagnosis based solely on quantitative measures (Fig. 1c) and excluding known confounding drivers (e.g., multi-parity, preexisting hypertension and gestational diabetes mellitus), all samples were collected by the same personnel in a single hospital, all libraries were prepared by a single researcher, and all sequencing was performed on a single instrument. Our libraries should thus represent highly coherent sets. Nonetheless, our study does have its own limitations. Because all but one PE subject delivered at ≤ 36 weeks gestational age, whereas all CTRL patients delivered closer to term (≥ 37 weeks), PE and CTRL placentas were not gestationally matched. Therefore, additional studies using placentas delivered for other medical reasons (e.g., preterm labor) will be needed to evaluate which gene expression changes, if any, are simply attributable to earlier gestational age in the PE subjects. As stated above, our study was also limited by small sample size particularly for the LO-PE group (n = 3). Future studies with adequate sample numbers are needed to fully characterize the transcriptional signature specific to LO-PE.

In addition to assessing protein-coding genes, we interrogated our PAS-Seq data with regard to non-coding and intergenic transcripts. While we found little evidence of differential long non-coding RNA expression in PE, our data do suggest a potential association between EO-PE and endogenous retroviral element activation. Thus it may be of interest to interrogate repeat-associated transcripts in existing PE placental RNA-Seq datasets^37,46^. Our PAS-Seq data also confirm upregulation of miR210 and downregulation of miR214 in PE^54^. Therefore, some gene expression differences associated with PE are likely driven by post-transcriptional mechanisms.

## Methods

### Ethics Statement

The study was approved by the Beth Israel Deaconess Medical Center Institutional Review Board (IRB), #2008P-000061. All subjects provided written informed consent for use of placental and blood samples for research.

### Human subjects

Placentas were collected from normal(CTRL) and PE subjects (Table 1) in accordance with all institutional policies and with approval of the institutional review board at the Beth Israel Deaconess Medical Center (Boston, MA, USA). The diagnosis of PE was based on the updated criteria of American College of Obstetrician and Gynecology Task force on Hypertension in Pregnancy^89^. Patients with history of diabetes, chronic hypertension, renal disease or multiple gestations were excluded. Pre-delivery maternal plasma samples were used to measure circulating sFlt1 and PlGF using commercial ELISA kits (R & D systems, MN) as described elsewhere^90^.

### Tissue collection, storage and RNA isolation

We excised placental biopsies (2 × 2 cm) without basal and chorionic plates and wiped with cotton gauze to remove blood and debris. The villous tissue, the spongy layer that is in between the decidua (maternal side) and the chorion (fetal side) was collected since it is the main source of sFlt1^34^. These tissue samples were flash-frozen in liquid nitrogen within 30 min of placental delivery.

### PAS-Seq library preparation and sequencing

For preparation of PAS-Seq libraries, we adapted a previously published method for making libraries from RNA fragments^91^; (Fig. 2a). Total RNA integrity was first confirmed by agarose electrophoresis (data not shown) and then polyA + RNA enriched by oligoT hybridization. PolyA + RNA samples were then fragmented to 60–80 nt via chemical hydrolysis and reverse transcribed with one of twelve anchored oligoT oligonucleotides containing forward and reverse Illumina sequencing primer sites separated by a hexa-ethyleneglycol spacer (Sp18) linker. At the 5′ end, each oligonucleotide began with 5′p-GG to promote ligation^91^, followed by 5 random nucleotides (unique molecular index, UMI) to enable PCR duplicate removal. Each primer also harbored a unique 5 nt Hamming barcode (BC), allowing for sample multiplexing. Following cDNA circularization with CircLigase I, libraries were PCR amplified (12–14 cycles) and subjected to single end 100 nt sequencing on the Illumina HiSeq platform in the UMass Medical School Deep Sequencing Core.

### PAS-Seq Bioinformatics analysis

After confirming overall library quality with FASTQC (http://www.bioinformatics.babraham.ac.uk/projects/fastqc/, S.Andrews, 2011), we removed duplicate reads with the same UMI. We then used Cutadapt^92^ to trim residual adaptor/spacer sequences and poly-A stretches from read 3′ ends and mapped the reads to hg19 reference genome using Bowtie2 (with parameters –m –best –p4). Only uniquely mapping reads with high quality scores (>20) were used for further analysis. Such reads represented 70–85% of total reads for all libraries.

We defined the polyadenylation cleavage site as the base closest to the poly-A stretch captured within read sequence, thus, very last base at the 3′-end site after the trimming. Based on this, we calculated read coverages of every genomic location considering only 3′-end of the aligned reads. This forms a sharp peak of read coverage around the cleavage sites with an average width 40nt (ranging between 1nt-120nt). We then clustered sites that are in proximity closer than 40nt window and summed the counts of clustered sites. In order to determine all possible polyadenylation sites, which might possibly differ from sample to sample, we pooled all candidate locations of all samples and reiterated the clustering using the same window length. Since genomic DNA containing poly-A stretches can be hybridized and pulled with the oligo-T primers, aka internal priming, we used a Naïve Bayes classifier^93^ based software to determine the likelihood of all sites being false priming sites. In addition to filtering internal priming locations based on this, we also removed background noise caused by widespread base level read alignments by fitting the count distribution of each gene to Poisson distribution. After determining precise PA locations, we then calculated expression/read counts of every PAS for each sample and annotated using Gencode v19. We also marked known polyA sites with PolyA DB (http://exon.umdnj.edu/polya_db/). For differential gene expression tests, we used sum of all PAS counts of each gene and calculated library sizes based on this. The raw read counts for genes were used as an input for differential gene expression analyses using DESeq. 2^38^ in R (https://www.R-project.org). The default normalization using ‘estimateSizeFactors’ function was used. Adjusted p-values were calculated using the Benjamini and Hochberg method^94^. In order to determine significance levels of alternative polyA site switches, we constructed a 2 by 2 contingency table composed of mean normalized read counts of one site and the mean of rest of the sites, if there are multiple, in each of the condition. We tested the significance with Chi-square test for given site and iterated through all sites of the gene. We then corrected p-values for multiple hypotheses testing using Benjamini and Hochberg method^94^.

## Electronic supplementary material

Supplementary Table S1

Supplementary Table S2

Supplementary Table S3

Supplementary Table S4

Supplementary Table S5

Supplementary Table S6

Supplementary Table S7

Supplementary Table S8

Supplementary Table S9

Supplementary Table S10

Supplementary Table S11
